# A deep learning-based method for grip strength prediction: Comparison of multilayer perceptron and polynomial regression approaches

**DOI:** 10.1371/journal.pone.0246870

**Published:** 2021-02-11

**Authors:** Jaejin Hwang, Jinwon Lee, Kyung-Sun Lee

**Affiliations:** 1 Department of Industrial and Systems Engineering, Northern Illinois University, DeKalb, IL, United States of America; 2 Department of Mechanical Engineering, Texas A&M, College Station, TX, United States of America; 3 Department of Industrial Health, Catholic University of Pusan, Busan, Republic of Korea; Ton Duc Thang University, VIET NAM

## Abstract

The objective of this study was to accurately predict the grip strength using a deep learning-based method (e.g., multi-layer perceptron [MLP] regression). The maximal grip strength with varying postures (upper arm, forearm, and lower body) of 164 young adults (100 males and 64 females) were collected. The data set was divided into a training set (90% of data) and a test set (10% of data). Different combinations of variables including demographic and anthropometric information of individual participants and postures was tested and compared to find the most predictive model. The MLP regression and 3 different polynomial regressions (linear, quadratic, and cubic) were conducted and the performance of regression was compared. The results showed that including all variables showed better performance than other combinations of variables. In general, MLP regression showed higher performance than polynomial regressions. Especially, MLP regression considering all variables achieved the highest performance of grip strength prediction (RMSE = 69.01N, R = 0.88, ICC = 0.92). This deep learning-based regression (MLP) would be useful to predict on-site- and individual-specific grip strength in the workspace to reduce the risk of musculoskeletal disorders in the upper extremity.

## 1. Introduction

Grip strength is an important measure to assess hand functionality and work capacity. In occupational settings, grip strength is commonly needed for using hand tools and operating the equipment in manufacturing, healthcare, and service industries [1–4]. Grip strength can be used for designing hand tools and workstations to reduce the risk of work-related injuries including carpal tunnel syndrome [5,6]. Maximum grip strength information could identify the work capacity, and it can be used for assessing the risk of work injuries [7]. For instance, if the work demand (load) is exceeding the grip strength (i.e., work capacity), it could increase the risk of work injuries.

Grip strength is known to be affected by many factors including gender, handedness, hand dimension, posture, and population [8–12]. Previous studies showed that males tended to have greater grip strength than females [9,13,14]. The dominant hand’s grip strength was higher than the non-dominant hand, but also fatigued more quickly [15,16]. Varying postures of the elbow and shoulder, and sitting/standing significantly affected the grip strength [9,17]. Caucasians tended to show greater grip strength than Asians [18,19].

A predictive model of the grip strength could be useful to industrial design and to prevent overexertion or overuse injuries. Previous studies mainly used the regressions models to predict the grip strength [9,20–23]. Although these models showed good predictive power, the linear relationship between the grip strength and demographic and anthropometric data of human subjects was assumed. However, it is likely that nonlinear relationship could exist between the grip strength and input variables, so non-linear or complex modeling could improve the predictive power of the grip strength [24].

Machine learning has been successful in various fields such as computer vision processing with Convolutional Neural Network (CNN) [25,26], time series prediction with Long Short-term Memory (LSTM) [27,28], and regression analysis with Multilayer Perceptron (MLP) [29–31]. MLP is a neural network with one or more hidden layers between the input layer and the output layer. MLP is able to classify nonlinear data through several hidden layers and nonlinear activation functions such as ReLU and tanh. In particular, regression analysis using MLP does not require the assumption regarding the statistical relationship between independent and dependent variables. Therefore, MLP has been widely used as an algorithm for regression in various fields [32–34].

Recently, the application of machine learning in the field of ergonomics is increasing. Several studies predicted human hand gestures by analyzing EMG data [35,36] or estimated a three-dimensional posture based on images using a CNN [37,38]. While deep learning studies predicting gestures and three-dimensional postures have been actively conducted, relatively few studies have investigated to predict grip strength. A previous study applied the neural network to estimate the grip strength for Malaysian industrial workers [24]. They considered the hand dimensions, age, wrist circumference, and weight as input variables, and their predicted grip strength was not significantly different from actual measurements. A recent study employed the deep neural networks classifiers to estimate and classify varying grip strength exertion levels [39]. They used facial video and photoplethysmogram data as input variables and resulted in 87–96% accuracy of classifications. Although these previous studies showed promising and robust results, the experimental postures were limited to the neutral forearm and wrist postures in sitting. Additional data covering a variety of postures-related grip strength would be useful to explore the functionality of the model. In addition, a different types of deep learning algorithms could be explored to determine whether the new approach produces more predictive power than existing approaches.

Our core research interest was whether the new methodology using deep learning showed higher accuracy in predicting the grip force than the existing regression analysis. The objective of this study was to predict grip strength using a deep learning-based regression (MLP) by considering a variety of postures, demographic and anthropometric information of human participants. In addition, we compared the performance of a deep learning-based regression (MLP) with the polynomial regressions (linear, quadratic, and cubic). Different combinations of input variables were considered to determine the optimal variable combination to produce the highest accuracy of the regression.

## 2. Methods

### 2.1. Participants

A total of 164 young adult university students (100 males and 64 females) were recruited for this study. The eligible criterion of a participant was no history of musculoskeletal disorders in the past 3 years. All participants were right-handed and their average age (standard deviation) was 22.8 (2.1) years. The mean (standard deviation) of height and weight was 170.5 (7.2) cm and 67.8 (10.0) kg, respectively. This study protocol was approved by the Institutional Review Board (Catholic University of Pusan Institutional Review Board-2020-026).

### 2.2. Experimental protocol

Upon the arrival and written consent of the approval, participants’ demographic information (gender, age, height, and weight) were collected. Hand dimensions (bilateral hand width and length) were measured by the researcher using Martin’s anthropometer (GPM Model 113, Switzerland). A total of 36 trials was randomly assigned to the participant, which was a combination of hand (left and right), lower body postures (sitting and standing), shoulder flexion angles (0°, 90°, and 180°), and forearm postures (pronation, supination, and neutral). Participants were instructed to exert maximal isometric grip strength for 3 seconds. This exertion was repeated 3 times with 2-minute breaks between trials. Based on that, the average maximum grip strength was calculated.

The adjustable Jamar handle dynamometer (Model J 00105, Lafayette Instrument Company) was used to measure the peak grip strength. The handle position of the dynamometer was set between the second and third handle positions based on each participant’s preference, which was adopted from the American Society of Hand Therapists for routine testing [40]. For the sitting posture, an adjustable chair was provided to fit each participant’s sitting height.

### 2.3. Data analysis

In order to estimate the grip strength, two approaches were considered: polynomial regression and deep learning-based regression (MLP). Polynomial regression is a statistical method in which the relationship between the independent variable *x* and the dependent variable *y* is modeled as an n^th^ degree polynomial in *x* [41]. If the order *d* of polynomial regression is large, the regression curve becomes too flexible and the performance decreases. Thus, we utilized quadratic and cubic as order *d*. MLP is one of the methods widely used for regression analysis in deep learning. It is able to learn the relationship between the data through multiple hidden layers.

**Fig 1:**
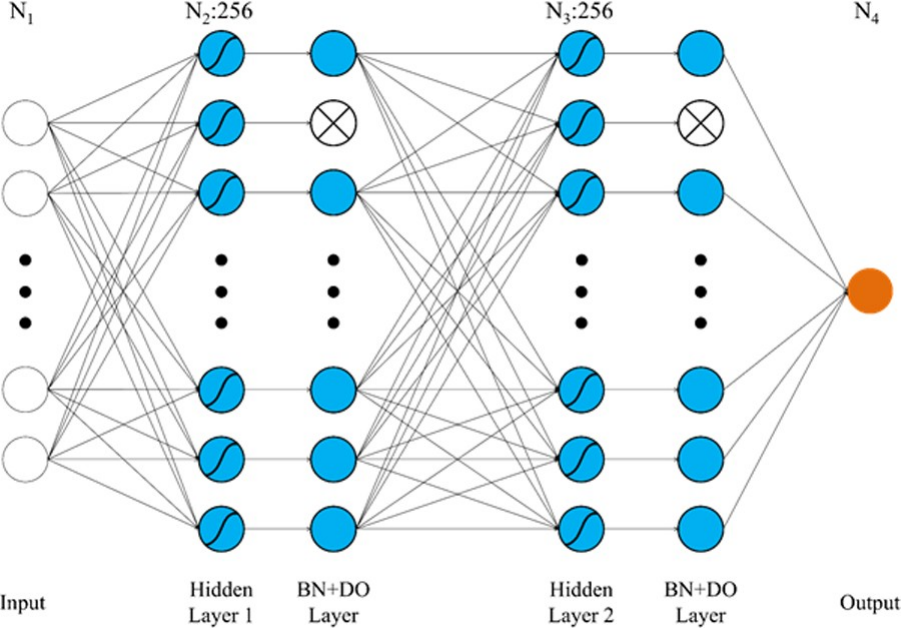
The neural network architecture. BN = batch normalization. DO = dropout.

Fig 1 shows our neural network architecture. The network consisted of an input layer, two hidden layers, two batch normalization (BN) and dropout (DO) layers, and an output layer. The input layer depends on the number of input variables (N_1_). The number of two hidden layers node was N_2_ (256) and N_3_ (256), respectively. Also, it utilized the tanh activation function to acquire nonlinearity. The BN is a method used to make faster learning and more stable by independently normalizing features [42]. The DO blocks the signal going to the node of the next layer to prevent overfitting for training data [43]. The dropout ratio value was set as 0.2. The output layer resulted in grip strength.

The dataset of 164 participants was divided into 148 for training (90%) and 16 for testing (10%). Given the high variation of grip strength among individuals, we chose 90% of data to be trained to increase the prediction performance of the regressions. The size of the dataset was considered insufficient to train machine learning. In order to augment the training data, we added Gaussian noise to grip strength within a reasonable range (*x*±20×*σ*) for augmentation. The Adam optimizer [44] was utilized with a learning rate of 0.001 for training. The batch size was 128, and all models were trained with the root mean square error (RMSE) as a loss function. In order to prevent overfitting, the model was trained for a maximum of 100 epochs, stopping early if the validation loss did not continue to decrease in 10 epochs. A robust scaler was employed to improve data stability and convergence speed in the optimization process. Robust scaler is a method that minimizes the influence of outliers and suitable for datasets with large differences in grip strength between individuals. Also, the K-fold cross-validation method was used to evaluate the performance of a prediction model in an unbiased manner. This method is known to be less sensitive to randomly segmented training datasets and is effective in preventing overfitting for small datasets [45]. K-fold cross-validation divided all the data into k groups of data of equal sizes. One group is for verification and the other group for training. The validation group was selected from a different group each time the model was trained. Our network was developed with python 3.7 and Keras 2.1.

The polynomial regressions with varying degrees (linear, quadratic, and cubic), and MLP regression were applied to a different combination of input variables. Table 1 illustrates a various predictive model that consists of different combination of variables based on the hand anthropometry, demographic information, and upper extremities and lower body postures. Model 1 included all variables that we obtained. Model 2 only consisted of posture-related variables. Model 3 only considered bilateral hand dimensions (length and width). Model 4 covered demographic and basic information of participants. Lastly, model 5 included a combination of variables used in models 2 and 4.

**Table 1:** Variable combination of input variables for 5 predictive models.

	Model 1	Model 2	Model 3	Model 4	Model 5
Gender	√			√	√
Age	√			√	√
Height	√			√	√
Weight	√			√	√
Hand	√			√	√
Hand length	√		√		
Hand width	√		√		
Lower body posture	√	√			√
Upper arm posture	√	√			√
Forearm posture	√	√			√

Note: Hand length and width indicate bilateral hand dimensions.

Polynomial regressions with varying degrees (linear, quadratic, and cubic) were employed for each combination of variables based on a training data set (148 participants; 90% of the total data). The regression equation of each model was applied to the test data set (16 participants; 10% of the total data) to validate the model.

For the performance measures to compare and validate different regressions, root mean squared error (RMSE), Pearson’s correlation coefficient (R), and intraclass correlation coefficients (ICC) were computed [46]. For the ICC values, less than 0.40 was poor; between 0.40 and 0.75 was good, and greater than 0.75 was considered excellent [47]. Original grip strength (reference) and predicted values from different models were compared in a plot.

## 3. Results

**Fig 2:**
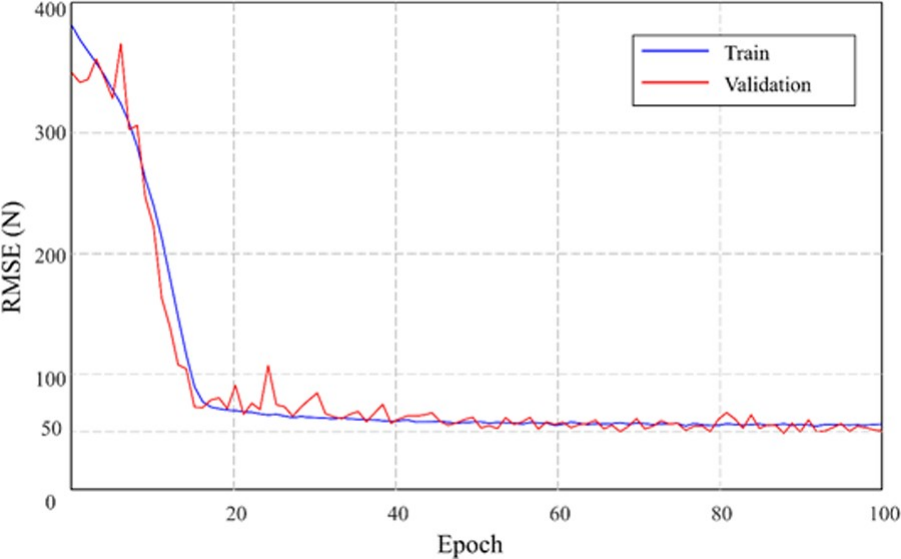
RMSE loss value curve for the epochs while running the MLP regression.

The mean and standard deviation of original grip strength (reference) of males and females were 457.6 (71.7) N and 223.6 (37.4) N, respectively. Fig 2 shows the loss value curve for the epochs while running the MLP regression. As the loss curve was shown, there was no big difference between the training curve and the validation curve. It was proven that the MLP regression was appropriately trained without under- and over-fitting. In order to determine the optimal number of hidden neurons, we tried several tests by changing the parameter values. When the number of hidden neurons was set as 32, 64, 128, 256, and 512, the resulting RMSE was 66.86, 64.61, 64.06, 62.76, and 63.39, respectively. Based on the results, the number of hidden neurons was set as 256.

**Fig 3:**
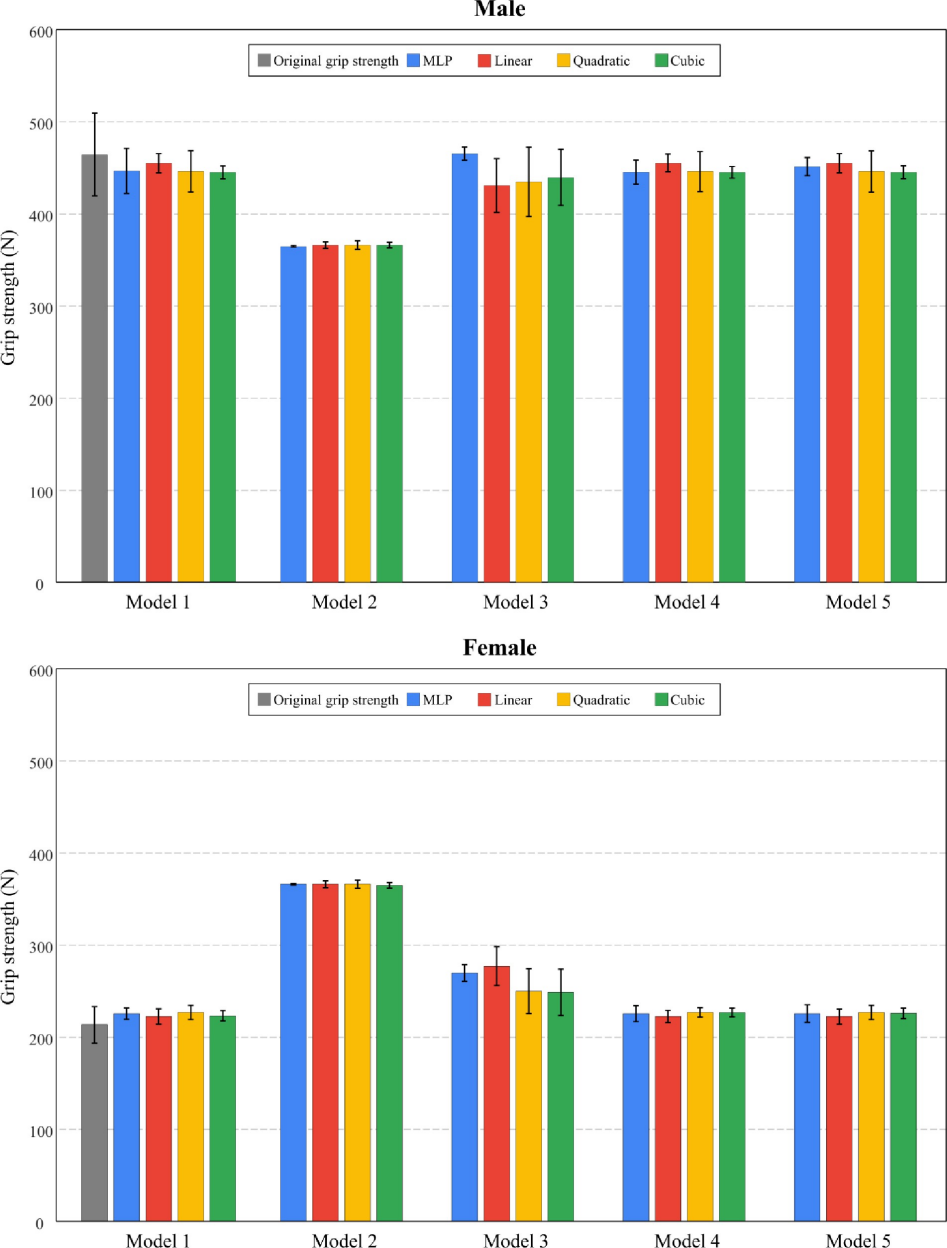
Mean and standard deviation of original grip strength (reference) and predicted values by gender from polynomial regressions (linear, quadratic, and cubic) and multi-layer perceptron (MLP) regression.

Comparison between the original values (reference) and predicted values from polynomial regressions with varying degrees and MLP regression were shown in Fig 3. Model 1 (considering all variables) showed the most comparable mean and standard deviation of grip strength relative to the original values. Model 2 (considering only posture-related variables) resulted in the greatest difference between original grip strength (reference) and predicted values.

**Fig 4:**
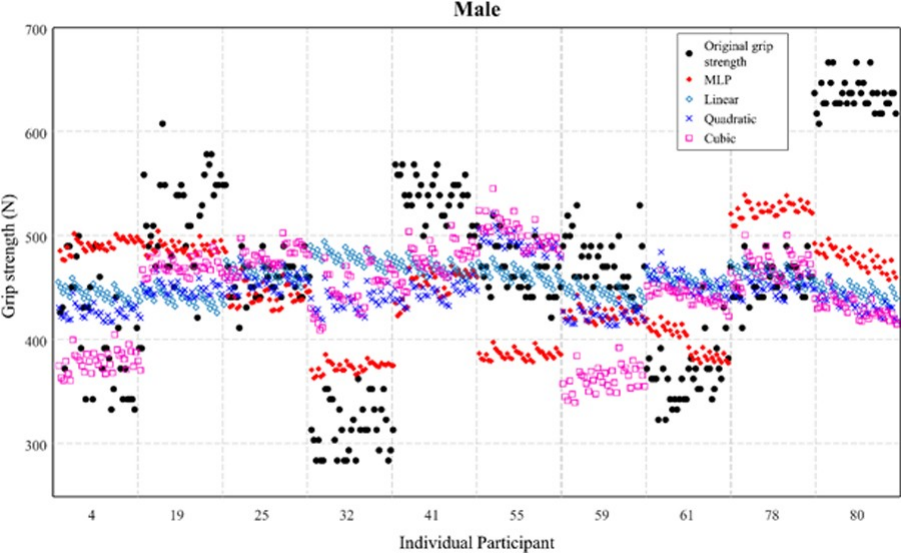
Predicted grip strength of males by multi-layer perceptron (MLP) and polynomial regressions (linear, quadratic, and cubic). Model 1 (including all variables) was considered.

**Fig 5:**
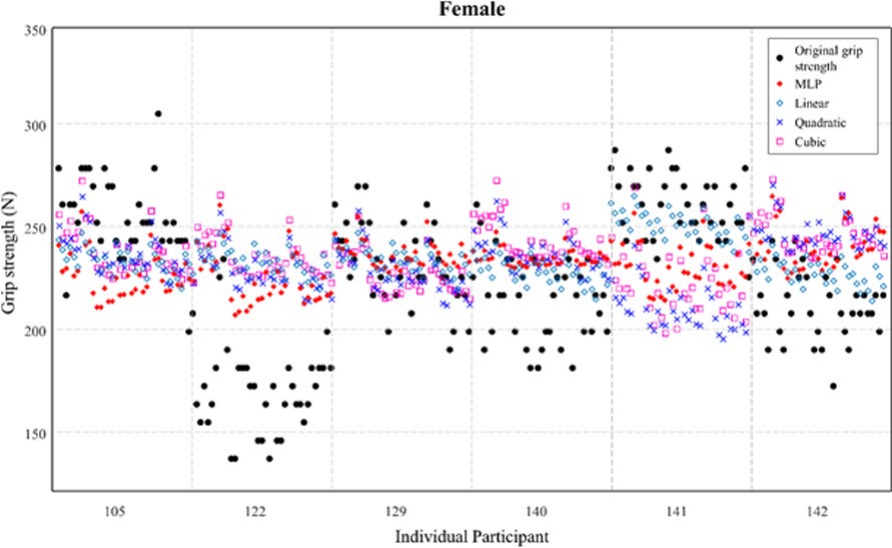
Predicted grip strength of females by multi-layer perceptron (MLP) and polynomial regressions (linear, quadratic, and cubic). Model 1 (including all variables) was considered.

Figs 4 and 5 illustrate the individual values of the original grip strength (reference) and predicted grip strength for males and females by polynomial regressions and MLP regression using model 1 (including all variables). Fig 4 showed that MLP regression revealed a better fit for original values compared to other polynomial regressions.

The performance of predicting grip strength by polynomial regressions and MLP regression were summarized in Table 2. For model 1, MLP showed higher performance than other polynomial regressions. For model 2, there were no practical differences between predictive regressions. For model 3, MLP regression showed greater performance than other polynomial regressions. For models 4 and 5, polynomial regressions tended to show greater performance than MLP regression.

**Table 2:** Comparison of the performances between the polynomial regressions (linear, quadratic, and cubic) and MLP regression.

	Regression	RMSE	R	ICC
Model 1	**MLP**	**69.01**	**0.88**	**0.92**
	Linear	79.03	0.83	0.90
	Quadratic	79.25	0.84	0.89
	Cubic	82.43	0.82	0.89
Model 2	MLP	142.69	0.03	0.001
	Linear	142.42	0.07	0.01
	Quadratic	142.40	0.06	0.01
	Cubic	142.37	0.07	0.02
Model 3	MLP	75.60	0.86	0.90
	Linear	93.76	0.76	0.83
	Quadratic	93.04	0.77	0.83
	Cubic	89.57	0.78	0.86
Model 4	MLP	85.69	0.80	0.87
	Linear	79.50	0.83	0.89
	Quadratic	79.69	0.83	0.89
	Cubic	82.56	0.82	0.88
Model 5	MLP	83.67	0.81	0.88
	Linear	79.03	0.83	0.90
	Quadratic	79.25	0.84	0.89
	Cubic	82.43	0.82	0.89

Note: MLP = multi-layer perceptron. Bold indicates the most effective regression.

**Fig 6:**
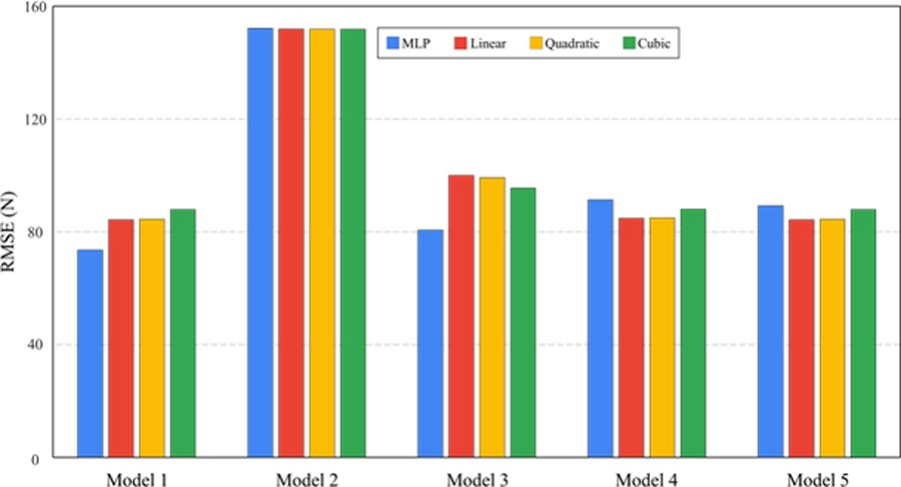
RMSE (N) of grip strength by MLP and polynomial regressions (linear, quadratic, and cubic).

Model 1 (including all variables) resulted in the lowest RMSE (mean: 77.4 N) (Fig 6). In overall, MLP regression showed lower RMES (91.3N) compared to other polynomial regressions (94.7 to 95.9N). Especially, MLP regression using Model 1 showed the lowest RMSE (69.0N) among different models (Fig 6).

## 4. Discussion

This study predicted grip strength using a deep learning-based regression (MLP) by considering a variety of postures, demographic and anthropometric information of human participants. To understand the robustness of the deep learning-based regression (MLP), the performance was compared with polynomial regressions (linear, quadratic, and cubic). Results showed that MLP regression exhibited higher performance than other polynomial regressions. The Model 1 (considering all variables) showed the most comparable mean and standard deviation of grip strength relative to the original values. Especially, MLP regression with Model 1 showed the lowest RMSE (69.0N) of grip strength.

In this study, the mean (standard deviation) grip strength of the male and female participants were 457.6 (71.7)N and 223.6 (37.4)N, respectively, which was comparable to the normative dataset of grip strength from Size Korea [48]. In the present study, the coefficient of variation (CV) of grip strength among males and females was 15.7% and 16.7%, respectively. This indicated consistent variability of grip strength, and they were within the range of CV (15.4 to 22.8%) in previous studies among US and Malaysian workers [24].

Overall, model 1 (including all variables) showed better performance than other models. For instance, RMSE of grip strength with model 1 was reduced by 3.7 to 65.0N compared to other models. Since the model 1 was fully trained by all variables, it revealed the highest performance of the model. This was consistent with the previous study showing that considering all variables resulted in the highest performance of MLP network model [49]. This pattern was more apparent in MLP regression rather than polynomial regressions.

Deep learning-based MLP regression showed greater performance than polynomial regressions except for a few models. MLP regression estimated the grip strength through the product of the weight and the sum of bias in several nodes of the hidden layer. Especially, complex network structure including two hidden layers, sufficient nodes and coefficients, and nonlinear activation function embedded in MLP regression resulted in better performance than polynomial regressions, which had a limited number of coefficients.

Among all different combinations of the regressions and models, MLP regression using model 1 showed the greatest performance for the prediction of grip strength. Model 1’s MLP showed the lowest RMSE of 69.01 N, which was 6.59 N lower than the second highest combination (MLP regression using model 3). As shown in Figs 4 and 5, it revealed the clear difference of prediction of individual grip strength between MLP and polynomial regressions. For instance, the prediction from polynomial regressions exhibited a small standard deviation of the grip strength (i.e., a narrow range of prediction), while MLP regression resulted in a comparable standard deviation with the original values. If all variables (demographic and anthropometric information, and postures) can be obtained in the field, MLP regression would produce the highest accuracy of the grip strength prediction. If the hand dimensions are only available data in the field, MLP regression would produce a slightly lower but still robust prediction of the grip strength.

**Fig 7:**
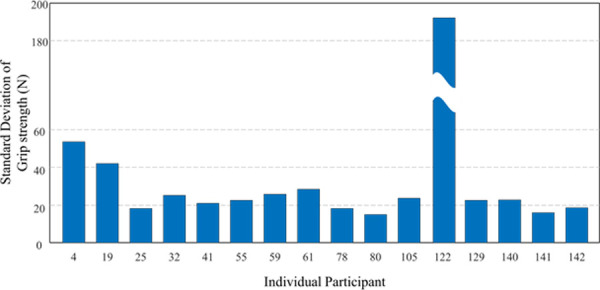
The standard deviation of grip strength (N) by the individual participant.

Although MLP regression using model 1 showed promising results (R = 0.88, ICC = 0.92), there was still a considerable amount of difference between the original and predictive values (RMSE = 69.01 N). This could be related to the variation of individual grip strength. As shown in Fig 7, certain participants showed much higher variability (participant 122: 185N) of exerting the maximum grip strength among different postural conditions compared to other participants. These few extreme data patterns could affect the learning process of MLP regression. In order to improve the performance of MLP regression, it would be necessary to collect a larger dataset containing a sufficient amount of extreme data characteristics.

Grip strength is an important measure to understand individual workers’ physical strength capability and designing a safe hand tool (e.g., optimal grip span) to reduce the risk of musculoskeletal disorders such as cumulative trauma disorders. Grip strength prediction developed in this study could be useful to determine the maximum grip strength in specific worksites without directly measuring the grip strength of industrial workers. For instance, based on the information of individual workers’ characteristics and frequently used postures in the workspace, the deep learning-based model could be applied to estimate the maximum grip strength.

There were several limitations noted in this study. First, this study only considered the maximum grip strength data of each participant. Time series data of grip strength by various postures may provide more information (e.g., time to reach peak value) to train the model and improve the model performance. Second, input variables considered in this study may not cover all possible variables associated with the grip strength. For instance, an individual’s muscle strength and other individual characteristics (e.g., fitness level) could be a great addition to improve the model performance. Although MLP regression showed promising results, we did not test other types of deep learning methods (e.g., convolutional neural network, or Long short-term memory network) due to the limitation of our data set (i.e., non-time series data). By collecting time series grip strength data, a future study could explore a wide range of deep learning-based methods.

## 5. Conclusion

This study applied the deep learning-based method (MLP regression) to accurately estimate the grip strength and compared the robustness of the regression with various polynomial regressions. The combinations of variables affected the performance of regressions significantly. By considering all variables including demographic and anthropometric information of individuals, and varying postures, MLP and polynomial regressions showed higher performance than other combinations of variables. Overall, MLP regression showed greater performance than polynomial regressions to predict grip strength. Especially, MLP regression considering all variables achieved the highest performance (RMSE = 69.01N, R = 0.88, ICC = 0.92). This study is regarded as the first study to apply the MLP model to predict grip strength and prove its effectiveness compared to existing regression analyses. The detailed structure and attribute information of the MLP model presented in this study can be used as an essential scientific value to enhance the model’s performance in the future. This deep learning-based method (MLP) could be useful to estimate the individual- and onsite- specific grip strength based on individual characteristics and frequently used postures in the certain workspace. The fact that the grip force can be predicted without direct measurement using the grip force device will be an advantage that can be practically used in the field. This information would be useful to properly design the hand tools and work arrangement to reduce the risk of musculoskeletal disorders. In future studies, the amount of the error in prediction due to the variation of the grip force could be further reduced by increasing the sample size. If additional variables such as muscle mass and fitness level of the participants are introduced, the model’s accuracy is expected to increase further.
